# Linking multi-level population dynamics: state, role, and population

**DOI:** 10.7717/peerj.13315

**Published:** 2022-05-12

**Authors:** Nao Takashina

**Affiliations:** Department of International Studies, The University of Tokyo, Chiba, Japan

**Keywords:** Lotka–Volterra model, McKendrick–von Foerster model, Ontogenetic diet shift, Population dynamics, Role shift, Species interactions, Individual variations

## Abstract

The dynamics of an ecological community can be described at different focal scales of the species, such as individual states or the population level. More detailed descriptions of ecological dynamics offer more information, but produce more complex models that are difficult to analyze. Adequately controlling the model complexity and the availability of multiple descriptions of the concerned dynamics maximizes our understanding of ecological dynamics. One of the central goals of ecological studies is to develop links between multiple descriptions of an ecological community. In this article, starting from a nonlinear state-level description of an ecological community (generalized McKendrick–von Foerster model), role-level and population-level descriptions (Lotka–Volterra model) are derived in a consistent manner. The role-level description covers a wider range of situations than the population-level description. However, using the established connections, it is demonstrated that the population-level description can be used to predict the equilibrium status of the role-level description. This approach connects state-, role-, and population-level dynamics consistently, and offers a justification for the multiple choices of model description.

## Introduction

The dynamics of an ecological community can be described at different focal scales of the species, such as individual states or the population level (Banasiak & Lachowicz, 2014; Lachowicz, 2011; Law, Murrell & Dieckmann, 2003). More detailed descriptions of ecological dynamics offer a greater amount of information, but provide more complex models that are difficult to analyze. Adequately controlling the model complexity according to the question at hand maximizes our understanding of the dynamics of an ecological community. Establishing links between multiple descriptions of an ecological community is one of the central goals of ecological studies (Lachowicz, 2011; Nakazawa, 2015; Ovaskainen et al., 2014; Poisot, Stouffer & Gravel, 2015) because consistent connections offer a flexible choice of model and justify the ecological implications of the focal level deduced from the description of another level.

In the dynamics of an ecological community, species interactions characterize the level of description. For example, a population-level description is characterized by homogeneous interactions between two species, and each species is characterized as prey, predator, or competitor (Allesina & Tang, 2012; Mougi, 2012). At this level of discussion, the types of interactions can be classified according to the interaction compass, where the signs of the interaction coefficients reflect each possible type of interaction (*e.g*., (−, +) and (+, −) for predation and (−, −) for competition) (Haskell, 1949; Nathaniel Holland & DeAngelis, 2009). This offers a concise description of ecological dynamics *via* the Lotka–Volterra model (Murray, 2004), and has been a major driver of studies on ecological dynamics and stability (*e.g*., May, 1972; Mougi, 2012; Serván et al., 2018). Alternatively, in a state-level description, each individual is grouped into sets of states (*e.g*., age or weight), and the interaction compass is no longer useful; instead, infinitely many interaction strengths are required. The state-level description offers a detailed and more realistic description of a population because variations in ecological state are ubiquitous within a population (Bolnick et al., 2011; Poisot, Stouffer & Gravel, 2015); however, this description has a rather complex form.

This motivates an intermediate level of description for an ecological community at an ecologically relevant scale. A wide range of species exhibit an ontogenetic diet/trophic niche shift (Winemiller, 1989; Yang & Rudolf, 2010) with or without a drastic habitat shift (Takimoto, 2003). The ontogenetic shift may largely alter the interspecific interaction strength among individuals within a population, and these shifts can lead to multiple roles at different life stages of the individuals (Nathaniel Holland & DeAngelis, 2009; Yang & Rudolf, 2010). For example, in a fish community, larger individuals of the fish species prey on smaller individuals available in a particular space (Hartvig & Andersen, 2013), where individuals of the species have prey and predator roles at different life stages. The combination of role shifts can be any set of, for instance, prey, predator, or superior/inferior competitor. This role-level view may provide an intermediate description of an ecological community.

Size- or age-structured models (Hartvig, Andersen & Beyer, 2011; Hastings, 1987; Takashina & Fiksen, 2020) and physiologically structured population models (De Roos et al., 2008; Metz & Diekmann, 1986) are commonly used to integrate states into population dynamics. Although this offers more detailed descriptions of the population dynamics than a population-level description, it usually demands more assumptions and parameters. For instance, Hartvig, Andersen & Beyer (2011) developed a size-structured community model focusing on fish communities and highly mechanistic bases. In this framework, the size-dependent predation defined by a predator–prey mass ratio (Brose, 2010) determines the variable interaction strength. Although the model is described by mechanistic bases and is useful for discussing the coexistence of multiple species (Hartvig & Andersen, 2013; Zhang et al., 2013), the model complexity obscures a clear connection to existing population-level discussions. De Roos (1997) scaled up the physiologically structured juvenile and adult states to population-level dynamics. Their model can be complemented with non-structured resource dynamics, leading to the development of an extended model of the consumer–resource dynamics (*i.e*., (−, +) and (+, −) interaction types in the interaction compass) by Yodzis & Innes (1992). Alternative approaches starting from stochastic individual-level dynamics that scale to deterministic population-level dynamics have also been widely discussed (*e.g*., Banasiak & Lachowicz, 2014; McKane & Newman, 2004; Ovaskainen et al., 2014).

Given the important role of the Lotka–Volterra model in ecological studies that can capture various types of species interactions, as well as the importance of the ecological state in population dynamics, it is fundamental to ask how and when the state-level description of an ecological community are related to the Lotka–Volterra model. These insights provide a justification and highlight the limitations of the use of the Lotka–Volterra model, and give another potential description of the ecological community at different scales. In this article, I first introduce an *N*-species generalization of the nonlinear size-structured model (*i.e*., in the form of the McKendrick–von Foerster equation (Murray, 2004)), as discussed in Hastings (1987) and Nisbet & Gurney (1982), as a state-level description of an ecological community. By aggregating the states into new groups, I identify the connections between state-, role-, and population-level dynamics, where the population-level dynamics correspond to the Lotka–Volterra equation where role-level information can be carried over. The role-level model derived in the process of scaling-up the description of the ecological community is a new class of model that can describe a wider range of ecological communities than the population-level description can handle. The existing literature often discusses “stages,” such as juvenile or adult (*e.g*., De Roos, 1997; Hastings & Costantino, 1987; Hastings, 1987; Hin et al., 2011), rather than “roles” as an intermediate-level description. However, the role is considered in this article, as it captures a wider situation than the stage. For instance, there can be multiple roles within a single stage.

The goal of this study is to identify the links between multiple descriptions of ecological community dynamics, rather than to provide detailed investigations of each description. However, the role-level description has not been widely investigated in ecological studies, so I clarify some of the equilibrium properties of the role-level model in a simple situation. I demonstrate this by using the established connection: a simpler population-level description can predict the equilibrium states of a more complex role-level description. In the example, I consider three possible scenarios of role shifts as the result of an ontogenetic diet/trophic niche shift: (i) from inferior/superior competitor to superior/inferior competitor; (ii) from competitor to predator; and (iii) from prey to predator. The standard isocline method of population-level dynamics is used to discuss the equilibrium properties of the role-level dynamics caused by the links between different levels of the population dynamics. This is a convenient approach to analyze a more complex model using the knowledge of a simpler model while it may be only applicable to equilibrium properties and systems that converge to a single stable state.

The approach described in this article offers an opportunity to discuss population dynamics at multiple levels where the population-level dynamics correspond to the Lotka–Volterra model. The model complexity can be controlled, and further elaborations are straightforward. This also provides flexibility in the use of empirical data.

## Multi-Level Population Dynamics

### General concept of multiple descriptions of ecological community

**Figure 1:**
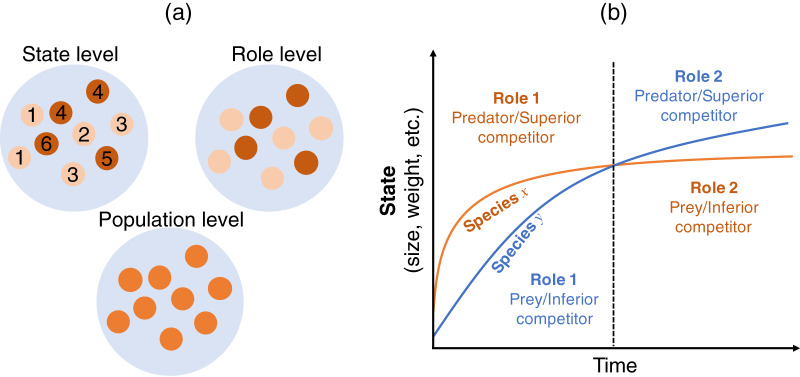
(A) Multiple views of a species at different levels. The state-level discussion focuses on groups of states, such as age or body size (labeled from 1–6), which have different roles in interactions with other species (*e.g*., competitor or predator); the role-level discussion no longer emphasizes the difference in states, but focuses on the group of roles (indicated by two colors); the population-level discussion does not distinguish among different roles within a population, but focuses on the emergent property of the species. (B) Conceptual diagram of role shift in individuals’ life histories. Consider two individuals from different species who are born at the same time and have interactions throughout their lifetimes. Species *x* is larger in size, weight, etc. than species *y* in the early life stage (left-hand side of the dashed line) and acts as the predator or superior competitor (role). This role changes in the later life stage (right-hand side of the dashed line), whereupon species *x* acts as the prey or inferior competitor. In reality, interactions occur among individuals of the two species in different life stages. The role of the individual in state *s* of species *i* over the interaction with state *s’* of species *j* is determined by the interaction strength $a_{i(s)j(s')}$. See the main text for a detailed explanation.

This section describes how the Lotka–Volterra model is derived as a population-level description from more detailed descriptions (state and role; Fig. 1A). I start with a state-level discussion of the situation presented in Fig. 1B, where the largest information can be incorporated. Then, we derive the role-level and population-level dynamics by scaling-up the focal level of the population dynamics, where we assume a simple situation where two species and two roles are considered. This approach is justified because the state-based dynamics contain information on the role- (if any) and population-level dynamics, but the opposite is not true. That is, scaling-up provides a simpler model but loses some information.

Notably, one can control the specificity (*i.e*., information) of the model by choosing what to scale up. Here, I demonstrate the role-level information is carried over to the Lotka–Volterra (population-level) model that contains larger information than the ordinary one where the interaction coefficients between species are fixed constant. The Lotka–Volterra model derived here provides an insight into the connection with nature where diverse interspecific interaction patterns are observed between species. The ordinary Lotka–Volterra model is readily recovered from the model by losing the information of role-shift (*i.e*., giving a fixed interaction strength). The role-level model contains the information of the Lotka–Volterra model, so the knowledge of the Lotka–Volterra model is applicable to the role-level model to some extent. By so doing, the connection to a different level of description becomes clear. I will demonstrate this in the following section.

### State-level dynamics

I begin with a state-level model that describes the *N*-species dynamics in which each individual of a species changes its state (*e.g*., age or weight) throughout its lifetime. The starting point is the common McKendrick–von Foerster equation (Murray, 2004). I extend this model by introducing the effects of intra- and inter-specific competition among the *N* species. The population dynamics of species *i* in state *s*, *n*_*i*_(*s*,*t*), can be described as

(1) $${{\partial {n_i}\left( {s,t} \right)} \over {\partial t}} + {{\partial g\left( s \right){n_i}\left( {s,t} \right)} \over {\partial s}} = - {d_i}{n_i}\left( {s,t} \right) - {n_i}\left( {s,t} \right)\sum\limits_{j = 1}^N {\int_0^\infty {{a_{i\left( s \right)j\left( {s'} \right)}}} } {n_j}\left( {s',t} \right)ds',$$

where *g*(*s*) is the rate of growth in state *s* on the state axis and *d*_*i*_ is the death rate of species *i*. *a*_*i*(*s*)*j*(*s*′)_ is the (nonnegative) interaction strength between state *s* of species *i* and state *s*′ of species *j*. The second term on the right-hand side accounts for the nonlinear effects of population dynamics, and is the fundamental difference from the size-structured community model (Hartvig, Andersen & Beyer, 2011) and physiologically structured population model (De Roos et al., 2008; Metz & Diekmann, 1986), in which nonlinearity appears in the term *g*(*s*) where, for instance, predation effects are incorporated (*i.e*., these are quasilinear equations). Note that Eq. (1) has the same form as the aforementioned models, except for the explicit nonlinearity, and it can be reduced to these models by removing the nonlinear term and defining *g*(*s*) accordingly. Equation (1) is complemented with the boundary conditions

(2) $${n_i}\left( {0,t} \right) = \int_0^\infty {{b_i}} \left( s \right){n_i}\left( {s,t} \right)ds + \sum\limits_{j = 1}^N {\int_0^\infty {\int_0^\infty {{{\hat b}_{i\left( s \right)j\left( {s'} \right)}}} } } {n_i}\left( {s,t} \right){n_j}\left( {s',t} \right)dsds',$$

where *b*_*i*_(*s*) is the birth rate of species *i* in state *s* and ${\hat b_{i\left( s \right)j\left( {s'} \right)}}$ is the birth rate of species *i* in state *s* as the result of predation on species *j* in state *s*′. Note that a cannibalistic interaction can be considered by setting ${\hat b_{i\left( s \right)i\left( {s'} \right)}} > 0$. These values are described as

(3a) $${b_i}\left( s \right) = \left\{ {\matrix{ {{\rm{non {-} negative}},} \hfill & {i\left( s \right)\ is\ matured} \hfill \cr {0,} & \hfill  {otherwise} \hfill \cr } } \right.,$$

(3b) $${\hat b_{i\left( s \right)j\left( {s^{\prime}} \right)}} = \left\{ {\matrix{ {\rm{non {-} negative}} \hfill & {i\left( s \right)\ preys\ on\ j\left( {s^{\prime}} \right)} \hfill \cr {0,} \hfill &  {otherwise} \hfill \cr } } \right.$$

where the notation *i*(*s*) indicates species *i* in state *s*. To describe the two-species role-level dynamics in the main text, the number of species is fixed to two, denoted as *n*_*x*_ and *n*_*y*_, respectively, instead of *n*_*i*_.

This is an *N*-species generalization of the nonlinear size-structured model discussed in Hastings (1987) and Nisbet & Gurney (1982).

### Role-level dynamics

The state-level model can be scaled-up to describe the dynamics of different roles in each species, and a variable corresponding to each role can be used to obtain the role-level dynamics. I assume that state development over time causes the ontogenetic diet/trophic niche shift in a well-mixed region; *i.e*., role shift. Here, I am interested in the general effect of this role shift on the population dynamics. For this purpose, I discuss the simplest possible situation, in which the rate of state growth is a constant *g*(*s*) = 1, the number of species is two (represented by *x* and *y*), and the number of roles is two, and only species *y* changes its role. *y*_1_ and *y*_2_ are non-overlapping role groups in population *y*, and *y*_1_ is assumed to be earlier stage than *y*_2_, where the role shift occurs in state *s*_1_. Integrating *n*_*x*_ and *n*_*y*_ accordingly, these are described as

(4) $$x = \int_0^\infty {{n_x}} \left( {s,t} \right)ds,{y_1} = \int_0^{{s_1}} {{n_y}} \left( {s,t} \right)ds,{y_2} = \int_{{s_1}}^\infty {{n_y}} \left( {s,t} \right)ds.$$

Hence, by integrating over state *s* in Eq. (1), the role-level model becomes (see Section “Derivation of the role-level dynamics”):

(5a) $${{dx} \over {dt}} = x\left( {{r_x} + \sum\limits_{{\cal X} \in \left\{ {x,{y_1},{y_2}} \right\}} {{a_{x{\cal X}}}} {\cal X}} \right),$$

(5b) $${{d{y_1}} \over {dt}} = {y_1}\left( {{r_{{y_1}}} + \sum\limits_{{\cal X} \in \left\{ {x,{y_1},{y_2}} \right\}} {{a_{{y_1}{\cal X}}}} {\cal X}} \right) - m{y_1} + \left( {{b_{{y_2}}} + {I_A}{a_{{y_2}x}}x} \right){y_2},$$

(5c) $${{d{y_2}} \over {dt}} = {y_2}\left( { - {d_{{y_2}}} + \sum\limits_{{\cal Y} \in \left\{ {{y_1},{y_2}} \right\}} {{a_{{y_2}{\cal Y}}}} {\cal Y} + \left( {1 - {I_A}} \right){a_{{y_2}x}}x} \right) + m{y_1},$$

where ${r_{\cal X}}$ is the growth rate of ${\cal X} \in \left\{ {x,{y_1},{y_2}} \right\}$, which can be either a species or a role (simply referred to as “type” hereafter). ${r_{\cal X}}$ can further be decomposed into ${b_{\cal X}} - {d_{\cal X}}$, where ${b_{\cal X}}$ and ${d_{\cal X}}$ are the birth rate and death rate of type ${\cal X}$, respectively. ${a_{{\cal X}{{\cal X}^\prime }}}$ represents the interaction strength between type ${\cal X}$ and ${{\cal X}^\prime } \in \left\{ {x,{y_1},{y_2}} \right\}$; a positive sign indicates that type ${{\cal X}^\prime }$ has a positive influence on the population of type ${\cal X}$, and *vice versa*. ${\rm{\sum}}{{}_{\cal X}}$ represents the sum over the variables. *m* is the transition rate from type *y*_1_ to *y*_2_. *I*_*A*_ is an indicator variable that is equal to 1 if *y*_2_ is a predator and equal to 0 otherwise.

Equation (5) has a similar form to the Lotka–Volterra-type model, but a major difference is that the birth event of role *y*_2_ increases the population number of role *y*_1_, and the transition rate *m* mediates the effect of the role shift.

The generalization of the role-level model is straightforward, and Section “Generalizations of the role-level model” describes generalized role-level models in which (i) species *y* has *n* roles; (ii) species *x* and *y* have *n*′ and *n* roles, respectively; and (iii) *N* species have (*n*_1_, … , *n*_*N*_) roles (*n*_*i*_ is the number of roles of species *i*). *Via* Eq. (5), various role-shift scenarios can be described: (i) from inferior/superior competitor to superior/inferior competitor; (ii) from competitor to predator; and (iii) from prey to predator. The specific forms of these models are provided in Section “Specific form of the role-level model”.

### Population-level dynamics

By further scaling-up the focal level of the population dynamics, the role-level model can be converted into the population-level model. This process aggregates the two roles *y*_1_ and *y*_2_ into a single variable. I introduce a new variable $\bar{y}$(*t*) = *y*_1_(*t*) + *y*_2_(*t*), and let *y*_1_(*t*) = (1 − *α*(*t*)) $\bar{y}$(*t*) and *y*_2_(*t*) = *α*(*t*) $\bar{y}$(*t*), where *α*(*t*) $\in$ [0,1] is the proportion of *y*_2_ in species *y* at time *t* (*i.e*., *α*(*t*) = *y*_2_(*t*)/(*y*_1_(*t*) + *y*_2_(*t*))). By summing Eqs. (5b) and (5c), Eq. (5) becomes

(6) $$\matrix{\hskip -1.7pc {{\displaystyle{dx} \over {\displaystyle dt}} = x\left( {{r_x} + {a_x}x + {a_{x\bar y}}\left( t \right)\bar y} \right),} \cr  {{{\displaystyle d\bar y} \over {\displaystyle dt}} = \bar y\left( {{r_{\bar y}}\left( t \right) + {a_{\bar y}}\left( t \right)\bar y + {a_{\bar yx}}\left( t \right)x} \right),} \cr }$$

where Eq. (6) has the form of the Lotka–Volterra model, but the parameters *$r_{\bar{y}}$*, $a_{x\bar{y}}$, $a_{\bar{y}x}$, and $a_{\bar{y}}$ are time-dependent because the role-level information is carried over. Therefore, Eq. (6) contains larger information than the usual Lotka–Volterra model. These parameters are described as

(7a) $${r_{\bar y}}\left( t \right) = \left( {1 - \alpha \left( t \right)} \right){r_{{y_1}}} + \alpha \left( t \right){r_{{y_2}}},$$

(7b) $${a_{x\bar y}}\left( t \right) = \left( {1 - \alpha \left( t \right)} \right){a_{x{y_1}}} + \alpha \left( t \right){a_{x{y_2}}},$$

(7c) $${a_{\bar yx}}\left( t \right) = \left( {1 - \alpha \left( t \right)} \right){a_{{y_1}x}} + \alpha \left( t \right){a_{{y_2}x}},$$

(7d) $${a_{\bar y}}\left( t \right) = {\left( {1 - \alpha \left( t \right)} \right)^2}{a_{{y_1}}} + \left( {{a_{{y_1}{y_2}}} + {a_{{y_2}{y_1}}}} \right)\alpha \left( t \right)\left( {1 - \alpha \left( t \right)} \right) + \alpha {\left( t \right)^2}{a_{{y_2}}}.$$

**Figure 2:**
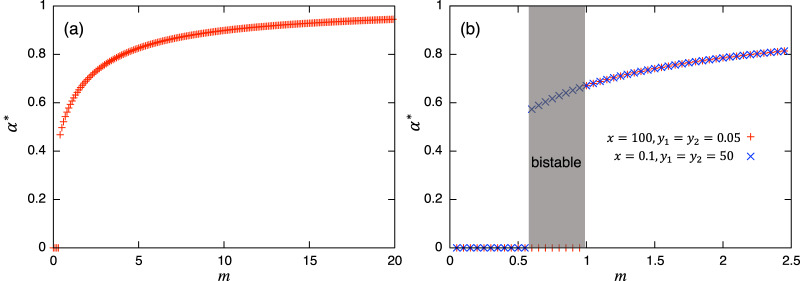
Relationships between the equilibrium value of $\alpha$ and *m*. In both panels, $\alpha^*=0$ suggests the extinction of species *y*. (B) is plotted using two initial conditions (corresponding to cross and plus) where bistability occurs. Except for *m*, the parameter values used are shown for the *a* in the first row of Table A.2, and for the *b* in the first row of Table A.4, respectively.

Values of *α* = 0 or 1 correspond to the usual (unstructured) Lotka–Volterra model, giving constant interaction coefficients. This Lotka–Volterra model can also be derived directly from Eq. (1) by scaling-up to a population level with a constant interaction strength (*a*_*xy*_, *a*_*yx*_). The magnitude of *α* is mediated by the transition rate *m*: a larger value of *α* is realized with a larger transition rate *m*, and *vice versa*. Note that each parameter in the role-shift model appears in the parameters in Eq. (7), and these mediate the effects of roles in the population-level model. While these coefficients change dynamically over time, *α*(*t*) converges to an equilibrium α* = y*_2_/(y*_1_ + y*_2_) if there is no periodic solution in the role-level dynamics. Hence, if there is a unique and stable nontrivial equilibrium, the stability of the equilibrium can be discussed *via* the ordinary analytical tools of the Lotka–Volterra model, such as isoclines. If there is a periodic solution such as the limit cycle or chaos, *α*(*t*) does not converge and this approach fails. There is ample literature about the stability issue of dynamical systems (*e.g*., Hirsch, Smale & Devaney, 2012; Strogatz, 2014), including the one in the Lotka–Volterra model (*e.g*., Hofbauer & Sigmund, 1998; Kot, 2001; Murray, 2004) where various methods for evaluating the stability of equilibrium can be found. Similarly, if the concerned system exhibits bistability, *α*(*t*) does not converge to a unique value, and again, the equilibrium analysis *via* the population-level model fails: the investigation of the role-level dynamics is necessary. Following analysis and numerical simulations suggest that the concerned role-level and population-level dynamics do not have a periodic solution, but these can exhibit bistability. To summarize the discussions above, Fig. 2 shows examples of the relationship between equilibrium value of *α* and *m*. Both panels show the increasing trends of α* as the transition rate *m* increases. Fig. 2B demonstrates the existence of bistability, and the presented equilibrium analysis cannot apply to this region.

## Analysis of Role-Level Dynamics

### Effect of role shift on interaction strength of population-level dynamics

**Figure 3:**
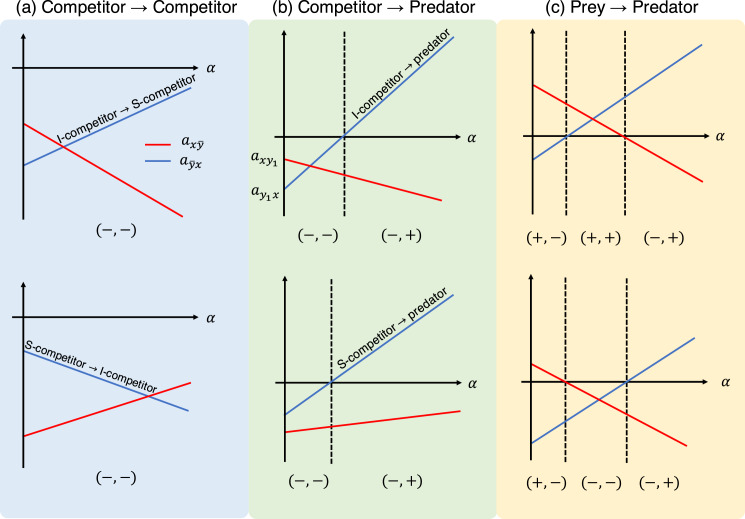
Interaction coefficients and their potential signs (shown at the bottom of each diagram) of the population-level model (Eq. (6)) with respect to the proportion parameter $\alpha$. Each panel shows different role-shift scenarios: (A) from competitor to competitor, (B) from competitor to predator, and (C) from prey to predator. In scenarios (A) and (B), competitors can be inferior or superior, as denoted by I-competitor and S-competitor, respectively. The parameters in the role-level model generate two different diagrams in (C). For example, the bottom figure is realized if $a_{y_1x}/(a_{y_1x}-a_{y_2x})< a_{xy_1}/(a_{xy_1}-a_{xy_2})$, which requires $|a_{x {\bar y}}| < |a_{{\bar y}x}|$.

As stated in the previous section, the parameters in the role-level model appear in the parameters of the population-level model (Eq. (7)). Hence, it is important to ask how the role shift affects the interaction strength between species ( ${a_{x\bar y}},{a_{\bar yx}}$) in the population-level model. From Eq. (7), it is apparent that ${a_{x\bar y}}$ and ${a_{\bar yx}}$ are monotonic increasing or decreasing functions of the proportional parameter *α*, depending on the signs of $a_{x{y_2}} - a_{x{y_1}}$ and $a_{{y_2}x} - a_{{y_1}x}$. Figure 3 shows schematic examples of the interaction coefficients in the population-level model as a function of the proportional parameter *α*. When the two roles are both competitors (*i.e*., inferior and superior competitors; Fig. 3A), the interaction coefficients ( ${a_{x\bar y}},{a_{\bar yx}}$) have the signs (−, −). Namely, the population-level model exhibits the features of the competition model. When the role shift is from competitor to predator (Fig. 3B), the signs of the interaction coefficients are either (−, −) or (−, +). The largest number of combinations is realized when the role shift is from prey to predator (Fig. 3C), which could produce the combinations (−, −), (−, +), (+, −), or (+, +), providing more diverse dynamics than the other role-shift scenarios. Curiously, the combinations (−, −) and (+, +) are not realized, given a single set of parameters, by changing the proportional parameter *α* (Fig. 3C, top and bottom).

### Coexistence and bistability of role-level and population-level dynamics

**Figure 4:**
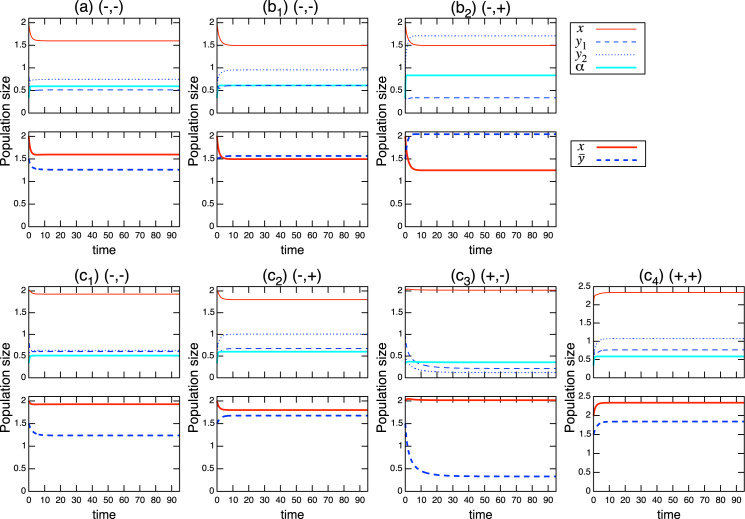
Coexistence of two species under the role-level (upper figure of each panel) and population-level (lower figure of each panel) dynamics with role-shift scenarios: (A) from inferior competitor to superior competitor; (B) from competitor to predator; and (C) from prey to predator. Scenarios (B) and (C) are further distinguished ( $\rm B_1, \ \rm B_2, \ \rm C_1, \ \rm C_1, \ \rm C_3, \ and \ \rm C_4$) based on the signs of the interaction parameters $(a_{x {\bar y}}, a_{{\bar y}x})$, where the signs are indicated in each panel. The parameter values used in the role-level dynamics are listed in Table A.2. The parameter values of the population-level dynamics can be readily obtained from Eq. (7) and the equilibrium proportion of $y_2\alpha^*$, in the corresponding role-level dynamics.

**Figure A.1:**
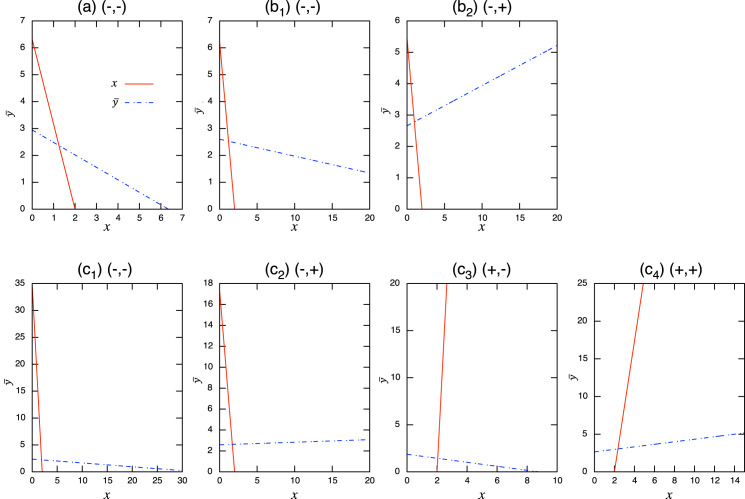
Isoclines of population-level model at equilibrium with role-shift scenarios from: (A) inferior competitor to superior competitor; (B) competitor to predator; and (C) prey to predator. The intersection of the two lines is the nontrivial equilibrium. Each panel corresponds to the population-level dynamics in Fig. 4.

Using the interaction coefficients in the population-level model, I now investigate the role- and population-level dynamics. In two-species population models, analysis *via* isoclines provides an outline of the dynamics (Murray, 2004). However, in the population-level model, the parameters in Eq. (7) vary with time, and ordinary isocline analysis is not directly applicable. Instead, I focus on the equilibrium properties of the dynamics, which can be determined by the configuration of isoclines in equilibrium. The equilibrium isoclines of the population-level model (Eq. (6)) are given by

(8) $$\matrix{ {}  {\bar y = - {\displaystyle 1 \over {\displaystyle a_{x\bar y}^*}}\left( {{r_x} + {a_x}x} \right),} \cr {}  {\bar y = - {\displaystyle 1 \over {\displaystyle a_{\bar y}^*}}\left( {r_{\bar y}^* + a_{\bar yx}^*x} \right),} \cr }$$

where * denotes equilibrium. Using these isoclines, it is straightforward to obtain the coexistence condition of the population-level dynamics (see Fig. A.1). For example, when the role shift is from inferior competitor to superior competitor and the interaction coefficients are ${a_{x\bar y}}\left( t \right) < 0$ and ${a_{\bar yx}}\left( t \right) < 0$, the coexistence condition is $a_{\bar yx}^*/{a_x} < r_{\bar y}^*/{r_x} < a_{\bar y}^*/a_{x\bar y}^*$. The full list can be found in Table A.1 and these are numerically verified. Examples of population-level dynamics and the corresponding role-level dynamics are presented in Fig. 4 for the case in which two species coexist. The parameter values used in the role-level dynamics are listed in Table A.2. These numerical calculations confirm that the role- and population-level dynamics are consistent in terms of the equilibrium values of two species.

**Figure 5:**
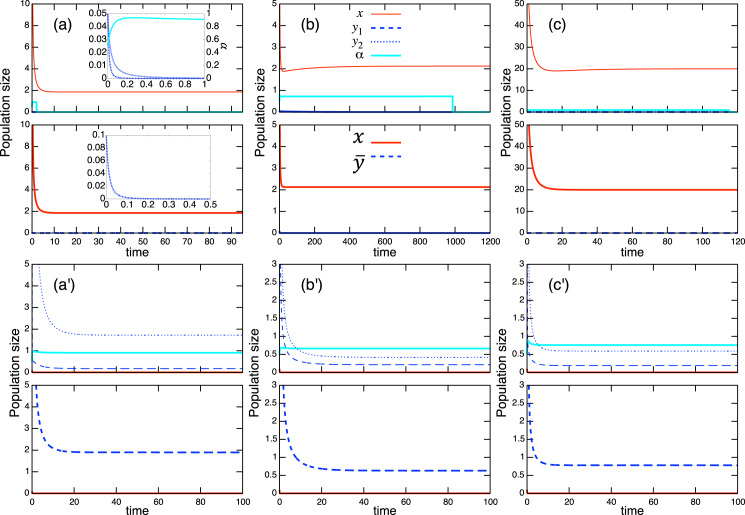
Bistability of the role-level (upper figure of each panel) and corresponding population-level (lower figure of each panel) dynamics with role-shift scenarios: (A, B′) from inferior competitor to superior competitor; (B, B′) from competitor to predator; and (C, C′) from prey to predator. The upper (A–C) show the situation in which species *y* becomes extinct (with the initial condition $x=100,\ y_1=y_2=0.05$) and the bottom (A′–C′) show that species *x* becomes extinct (with the initial condition $x=0.1, \ y_1=y_2=50$). I set $\alpha=0$ if $y_1+y_2<10^{-4}$. Inset figures in (A) show dynamics of species *y* and $\alpha$ near the initial states. The parameter values used in the role-level dynamics are listed in Table A.4. For the population-level dynamics, the parameter values are readily obtained from Eq. (7) and the equilibrium proportion of *y*_2_, $\alpha^*$, in the corresponding role-level dynamics.

**Figure A.2:**
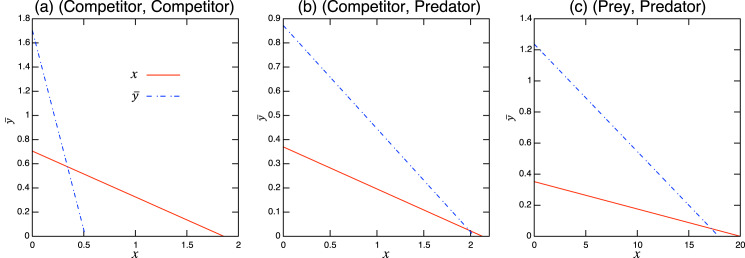
Isoclines at *x*-free equilibrium of the population-level model under bistability conditions with role-shift scenarios from: (A) inferior competitor to superior competitor; (B) competitor to predator; and (C) prey to predator. The intersection of the two lines is the nontrivial equilibrium. Each panel corresponds to the population-level dynamics in Fig. 5.

Considering the possible signs of the interaction coefficients in the population-level model (Fig. 3), bistability can occur in all role-shift scenarios. In this case, analysis *via* the equilibrium isoclines immediately provides the necessary conditions. The conditions for bistability are listed in Table A.3. Figure 5 shows the role-level and population-level dynamics under bistability conditions. The parameter values used in the role-level dynamics are listed in Table A.4. The upper figures (Figs. 3A–3C) show that species *x* excludes species *y*, while the lower figures (Figs. 3B′ and 3C′) show the opposite situation. There is clearly an agreement between the role- and population-level dynamics at equilibrium. The equilibrium isoclines of the population-level model is shown in Fig. A.2.

As expected, the aggregation of roles into the population-level dynamics does not always work under bistability conditions, because the values of the proportional parameter *α* differ in the two stable states. When the mismatch of equilibrium values between the role- and population-level dynamics is minor in simulations with randomly generated parameter values (ca. 0.24% over 10^5^ random parameter sets; see Section “Mathematical discussion of the role-level model” for more details), it is safe to analyze the role-level dynamics instead of the corresponding population-level effects.

## Discussion

Intraspecific state variations (*e.g*., age, weight) and multiple roles in species interactions (*e.g*., competitor and predator) within a population are ubiquitous (Winemiller, 1989; Yang & Rudolf, 2010). However, describing all these details comes at a significant cost of increased model complexity and analytical intractability. Thus, multiple methods of describing the ecological community are beneficial that allows us to simplify the discussion of ecological dynamics *via* a simpler description. Starting from the *N*-species dynamics in which each species has continuously varying states (Eq. (1)), the role-level (Eq. (5)) and population-level dynamics (Eq. (6)) are obtained by scaling-up to the focal level of description of the ecological community. In this sense, the role- and population-level models are not given models but derived from the more detailed state-level model. The population-level description corresponds to the Lotka–Volterra-type dynamics. We demonstrated that, even when a species has multiple interaction strengths in the interaction with another species, the equilibrium state can still be predicted by the Lotka-Volterra model. As the Lotka–Volterra dynamics have a nonlinear term, nonlinearity is also necessary in the state-level description to establish the connections. This approach is essentially an *N*-species generalization of the nonlinear size-structured model (*e.g*., Hastings, 1987; Nisbet & Gurney, 1982), but no explicit multi-species generalization or derivation of an intermediate-level description (*i.e*., role-level description) has previously, to the best of the author’s knowledge, been carried out.

I have demonstrated that an intermediate description is determined by the role between the interaction with another species, although alternative choices are also possible. For example, Hastings (1987) studied cannibalistic interactions between the eggs and larvae of Tribolium, and these life stages provide a better choice for the intermediate description. It is also reasonable to choose life stages for fish species and, in fact, state-level, life stage-level, and population-level discussions can be independently used (*e.g*., Clark, 2010; Micheli et al., 2004; Plank, 2017). These choices should be made based on the ecological question being considered.

The simplest situation was examined as a means of establishing the links between multi-level population dynamics in which species *y* has two roles, but it is easy to extend this situation. For example, the number of species and roles are arbitrary (Section “Generalizations of the role-level model”), and a nonlinear predation rate, such as Holling’s type II functional response (Murray, 2004), is straightforward to incorporate. Additionally, more complex models (De Roos, 1997; Hartvig, Andersen & Beyer, 2011) can incorporate growth on the state axis as the result of energy intake. As these models are described in the form of the McKendrick–von Foerster equation (Eq. (1), but without the nonlinear term), most such elaborations can be incorporated into the proposed model.

By taking advantage of the consistent link to the Lotka–Volterra model, I next demonstrated a potential simplification for the stability analysis of the role-level model using a population-level (*i.e*., Lotka–Volterra model) discussion in the case of a unique and stable nontrivial equilibrium. This offers new insights into species interaction and stability where a species has multiple interaction strengths in the interaction with another species. For the coexistence of two species where one species has two roles (*e.g*., prey and predator), the signs of the interaction coefficients and the number of plausible combinations of signs in the population-level dynamics (Eq. (6)) differ between role-shift scenarios. For example, with a role-shift scenario from an inferior to superior competitor, the only plausible signs of the interaction coefficients are (−, −). In contrast, the combinations (−, −), (−, +), (+, −), (+, +) are possible with the role-shift scenario from prey to predator (Table A.1). Thus, the model is capable of handling more diverse species interaction strengths. The transition rate *m* mediates the effect of the role shift caused by an ontogenetic diet/trophic niche shift, and influences the interaction coefficients in the population-level description (Eq. (7)). A role shift influences the magnitude of the interaction strength arbitrarily and, therefore, the coexistence condition at the population-level. However, as in the role-shift scenario from prey to predator, more diverse coexistence scenarios than the classical Lotka–Volterra-type competition and prey–predator dynamics are possible. Wollrab, de Roos & Diehl (2013) demonstrated that an ontogenetic diet shift enhances the chance of predator-mediated coexistence in competing prey species. The timing of the ontogenetic shift can be adaptive, leading to stabilized consumer–resource dynamics (Takimoto, 2003). Bistability or alternative stable states are possible in the Lotka–Volterra competition model (Murray, 2004); this is often referred to as the priority effect (Ke & Letten, 2018), where historical contingency affects the assembly and function of ecological communities (Chase, 2003; Fukami, 2015). I have shown that the role-level dynamics can also induce bistability under all role-shift scenarios discussed, suggesting that the priority effect can prevail in nature in the presence of various species interactions.

The coexistence and bistability conditions at the population-level discussion are provided in Tables A.3 and A.4. The dynamics at the role and population levels for each case are displayed in Figs. 4 and 5. This suggests that the parameters in the role-level model map onto a sufficiently large parameter space of the population-level model at equilibrium. That is, given a set of parameters in the population-level model, one is likely to find parameter sets in the role-level model that again correspond to the initial parameters *via* the relationship of Eq. (7). This property simplifies the discussion: one can start with a (simpler) population-level discussion, and later find a connection to the (more complex) role-level discussion; this is how the parameter sets used in Fig. 5 were identified. A detailed mathematical discussion will facilitate this aspect.

Climatic change affects ontogeny and phenology, and alters species interactions (Thackeray, Jones & Maberly, 2008; Yang & Rudolf, 2010). A general insight into the climatic effects on interspecific interactions is essential, and is one possible application of the model. In the context of such models, climate change leads to an altered proportional parameter (*i.e*., the proportion of *y*_2_) *α* in the population-level model (or a different rate of growth of *g*(*s*) in Eq. (1) or transition rate *m* in Eq. (5)). This will cause a shift in the values of the coefficient parameters, or may even change their signs (Fig. 3). For example, assume that the role-shift scenario is from inferior to superior competitor (Fig. 3A: top), and climate change increases the rate of growth of species *y*, causing a larger transition rate *m* to an alternative role and a larger value of *α*. These effects will increase $a_{x\bar{y}}$ and decrease $a_{\bar{y}x}$, respectively (Fig. 3A: top). Using Fig. 3, it is possible to predict potential sign shifts in the interaction coefficients induced by climatic changes. For example, in the role-shift scenario from prey to predator (Fig. 3C), $\left( { - , + } \right) \leftrightarrow \left( { + , + } \right) \leftrightarrow \left( { + , - } \right)$ and $\left( { - , + } \right) \leftrightarrow \left( { - , - } \right) \leftrightarrow \left( { + , - } \right)$ are possible, whereas the shift between (+, +) and (−, −) cannot occur.

In this article, I have demonstrated the explicit links between different descriptions of an ecological community (state-, role-, or population-level). The model complexity is controlled by scaling-up the focal level of the description. This offers the opportunity to attack ecological questions using various models that describe the same phenomena, but at different scales.

## Appendix

### Mathematical discussion of the role-level model

#### Derivation of the role-level dynamics

The population dynamics of species *i* in state *s* (*e.g*., size, age), *n*_*i*_(*s*,*t*), are described by Eq. (1) in the main text.^1^ 1The generalization to a multiple-state model may be obtained by replacing *s* with **s** in Eq. (1), where ${\bf{s}} = (s_1, s_2 , \ldots, s_n)\in \cal{S}$ is a state vector corresponding to a set of states in the *n*-dimensional state space. Provided that *s*_1_, $\ldots$ , *s*_*n*_ are orthogonal, the second term on the left-hand side of Eq. (A.1) is described by the gradient operator. The connection to the role-level dynamics (and consequently the population-level dynamics) is demonstrated by considering the simplest possible situation with a constant rate of state growth *g*(*s*) = 1. Let the number of species be fixed to two, denoted by *n*_*x*_ and *n*_*y*_, respectively, instead of *n*_*i*_. Furthermore, assume that species *y* has a role-shift property and each role is described by *y*_1_ and *y*_2_, where the role shift occurs in state *s*_1_. Hence, the population size of these roles are described by $x = \int_0^\infty {{n_x}} \left( {s,t} \right)ds,\ \ {y_1} = \int_0^{{s_1}} {{n_y}} \left( {s,t} \right)ds$, and ${y_2} = \int_{{s_1}}^\infty {{n_y}} \left( {s,t} \right)ds$. Now, the interaction coefficients depend on the species or role (type, hereafter) ${\cal X} \in \left\{ {x,{y_1},{y_2}} \right\}$, rather than the state. Hence, the subscripts of interaction strength *a*_*i*(*s*)*j*(*s*′)_ in Eq. (1) are replaced with the label of the role.

Integrating both sides of Eq. (1) over all possible *s* $\in$ [0,*∞*) yields

(A.1a) $${{dx} \over {dt}} = {n_x}\left( 0 \right) - {d_x}x - x\sum\limits_{{\cal X} \in \left\{ {x,{y_1},{y_2}} \right\}} {{a_{x{\cal X}}}} {\cal X},$$

(A.1b) $${{d{y_1}} \over {dt}} = {n_y}\left( 0 \right) - {n_y}\left( {{s_1}} \right) - {d_{{y_1}}}{y_1} - {y_1}\sum\limits_{{\cal X} \in \left\{ {x,{y_1},{y_2}} \right\}} {{a_{{y_1}{\cal X}}}} {\cal X},$$

(A.1c) $${{d{y_2}} \over {dt}} = {n_y}\left( {{s_1}} \right) - {d_{{y_2}}}{y_2} - {a_{{y_1}}}y_1^2 - {y_2}\sum\limits_{{\cal X} \in \left\{ {x,{y_1},{y_2}} \right\}} {{a_{{y_2}{\cal X}}}} {\cal X},$$

where *n*_*x*_(0) and *n*_*y*_(0) represent the recruitment of species *x* and *y*, respectively, and $\sum {}_{\cal{X}}$ represents the sum over variables ${\cal X} \in \left\{ {x,{y_1},{y_2}} \right\}$. As an example, *n*_*x*_(0) is obtained from the integration of the second term on the left-hand side in Eq. (1) with *i* = *x*: $\int_0^\infty \partial {n_x}\left( {s,t} \right)/\partial sds = n\left( {\infty ,t} \right) - n\left( {0,t} \right) = - n\left( {0,t} \right)$. Under the assumption of homogeneity within a role (*i.e*., all individuals with a certain role are identical), *n*_*x*_(0) = *b*_*x*_*x* ( = *∫ b*_*x*_*n*_*x*_(*s*,*t*)*ds*) and *n*_*y*_(0) = *b*_*y*1_*y*_1_ + *b*_*y*2_*y*_2_ + *I*_*A*_*a*_*y*2*x*_*xy*_2_, where ${b_{\cal X}}$ is the birth rate of type ${\cal X}$ and *a*_*y*2*x*_ is the reproductive term under the predation of species *x* by type *y*_2_. Additionally, *I*_*A*_ is an indicator variable with a value of 1 if *y*_2_ is a predator role and a value of 0 otherwise. *n*_*y*_(*s*_1_) represents a shift from type *y*_1_ to type *y*_2_. This can be described as *my*_1_, where *m* is the transition rate. By summarizing these discussions, and noting that the net growth rate is described by ${r_{\cal X}} = {b_{\cal X}} - {d_{\cal X}}$, Eq. (A.1) corresponds to role-level dynamics defined in Eq. (5) in the main text. I define the coefficient matrix of Eq. (5) as

(A.2) $$\left( {\matrix{ {{a_x}} \hfill & {{a_{x{y_1}}}} \hfill & {{a_{x{y_2}}}} \hfill \cr {{a_{{y_1}x}}} \hfill & {{a_{{y_1}}}} \hfill & {{a_{{y_1}{y_2}}}} \hfill \cr {{a_{{y_2}x}}} \hfill & {{a_{{y_2}{y_1}}}} \hfill & {{a_{{y_2}}}} \hfill \cr {} \hfill & {} \hfill & {} \hfill \cr } } \right),$$

where ${a_{{\cal X}{\cal X}}}$ is denoted as ${a_{\cal X}}$ for brevity. The signs of the components of the coefficient matrices for the three situations of (i) inferior competitor to superior competitor, (ii) competitor to predator, and (iii) prey to predator become

(A.3) $$\left( {\rm{i}} \right)\left( {\matrix{ - \hfill & - \hfill & - \hfill \cr - \hfill & - \hfill & - \hfill \cr - \hfill & - \hfill & - \hfill \cr {} \hfill & {} \hfill & {} \hfill \cr } } \right),\left( {{\rm{ii}}} \right)\left( {\matrix{ - \hfill & - \hfill & - \hfill \cr - \hfill & - \hfill & - \hfill \cr + \hfill & - \hfill & - \hfill \cr {} \hfill & {} \hfill & {} \hfill \cr } } \right),\left( {{\rm{iii}}} \right)\left( {\matrix{ - \hfill & + \hfill & - \hfill \cr - \hfill & - \hfill & - \hfill \cr + \hfill & - \hfill & - \hfill \cr {} \hfill & {} \hfill & {} \hfill \cr } } \right).$$

**Table A.1:** Coexistence conditions in the population-level model (Eq. (6)).

Signs of ( $a_{x \bar{y}}, a_{ \bar{y} x}$)	Coexistence condition
Competitor (*y*_1_) → Competitor (*y*_2_)	
(i) (−, −)	$\displaystyle{{a_{\bar yx}^*} \over {{a_x}}} < {{r_{\bar y}^*} \over {{r_x}}} < {{a_{\bar y}^*} \over {a_{x\bar y}^*}},\left( {{r_x} > 0,r_{\bar y}^* > 0} \right)$
Competitor (*y*_1_) → Predator (*y*_2_)	
(ii.a) (−, −)	Same as (i)
(ii.b) (−, +)	$\left\{ \matrix{ {{r_{\bar{y}}^*} \over {{r_x}}} > {{a_{\bar{y}x}^*} \over {{a_x}}},\left( {{r_x} > 0,r_{\bar{y}}^* < 0} \right) \hfill \cr {{r_{\bar{y}}^*} \over {{r_x}}} < {{a_{\bar{y}}^*} \over {a_{x\bar{y}}^*}},\left( {{r_x} > 0,r_{\bar{y}}^* > 0} \right) \hfill \cr} \right.$
Prey (*y*_1_) → Predator (*y*_2_)	
(iii.a) (−, −)	Same as (i)
(iii.b) (−, +)	Same as (ii.b)
(iii.c) (+, −)	$\left\{ \matrix{ {{r_{\bar{y}}^*} \over {{r_x}}} < {{a_{\bar{y}}^*} \over {a_{x\bar{y}}^*}},\left( {{r_x} < 0,r_{\bar{y}}^* > 0} \right) \hfill \cr {{r_{\bar{y}}^*} \over {{r_x}}} > {{a_{\bar{y}x}^*} \over {{a_x}}},\left( {{r_x} > 0,r_{\bar{y}}^* > 0} \right) \hfill \cr} \right.$
(iii.d) (+, +)	$\left\{ \matrix{ {{a_{\bar{y}}^*} \over {a_{x\bar{y}}^*}} < {{a_{\bar{y}x}^*} \over {{a_x}}},\left( {{r_x} > 0,r_{\bar{y}}^* > 0} \right) \hfill \cr {{a_{\bar{y}}^*} \over {a_{x\bar{y}}^*}} < {{a_{\bar{y}x}^*} \over {{a_x}}}\;\;\;{{r_{\bar{y}}^*} \over {{r_x}}} > {{a_{\bar{y}x}^*} \over {{a_x}}},\left( {{r_x} > 0,r_{\bar{y}}^* < 0} \right) \hfill \cr {{a_{\bar{y}}^*} \over {a_{x\bar{y}}^*}} < {{a_{\bar{y}x}^*} \over {{a_x}}}\;\;\;{{r_{\bar{y}}^*} \over {{r_x}}} < {{a_{\bar{y}}^*} \over {a_{x\bar{y}}^*}},\;\;\left( {{r_x} < 0,r_{\bar{y}}^* > 0} \right) \hfill \cr} \right.$

**Table A.2:** Parameter values used in Fig. 4.

Panel	*b*_*x*_	*by*_1_	*by*_2_	*d*_*x*_	*dy*_1_	*dy*_2_	*a*_*x*_	$\boldsymbol a_{xy_1}$	$\boldsymbol a_{xy_2}$	$\boldsymbol a_{y_1x}$	$\boldsymbol a_{y_1}$	$\boldsymbol a_{y_1y_2}$	$\boldsymbol a_{y_2 x}$	$\boldsymbol a_{y_2 y_1}$	$\boldsymbol a_{y_2}$	*m*
*a*	1.2	0	1.2	0.2	0.1	0.1	−0.5	−0.1	−0.2	−0.2	−0.5	−0.1	−0.1	−0.1	−0.5	1.0
*b*_1_	1.2	0	1.2	0.2	0.1	0.1	−0.5	−0.1	−0.2	−0.2	−0.5	−0.1	0.1	−0.1	−0.5	1.0
*b*_2_	1.2	0	1	0.2	0.1	0.1	−0.5	−0.1	−0.2	−0.2	−0.5	−0.1	0.1	−0.1	−0.5	5.0
*c*_1_	1.2	0	1	0.2	0.1	0.1	−0.5	0.15	−0.2	−0.2	−0.5	−0.1	0.15	−0.1	−0.5	0.5
*c*_2_	1.2	0	1	0.2	0.1	0.1	−0.5	0.15	−0.2	−0.2	−0.5	−0.1	0.15	−0.1	−0.5	1.0
*c*_3_	1.2	0	1	0.2	0.1	0.1	−0.5	0.15	−0.2	−0.2	−0.5	−0.1	0.15	−0.1	−0.5	0.1
*c*_4_	1.2	0	1	0.2	0.1	0.1	−0.5	0.15	−0.2	−0.2	−0.5	−0.1	0.2	−0.1	−0.5	1.0

**Table A.3:** Bistability conditions in the population-level model (Eq. (6)).

Signs of ( $\boldsymbol a^*_{{x}{\overline y}}, a^*_{{\overline y} x}$)	Coexistence condition
Competitor (*y*_1_) → Competitor (*y*_2_)	
(i) (−, −)	$\displaystyle{{a_{\bar y}^*} \over {a_{x\bar y}^*}} < {{r_{\bar y}^*} \over {{r_x}}} < {{a_{\bar yx}^*} \over {{a_x}}}, \ \ \ \left( {{r_x} > 0,r_{\bar y}^* > 0} \right)$
Competitor (*y*_1_) → Predator (*y*_2_)	
(ii) (−, −)	Same as (i)

Prey (*y*_1_) → Predator (*y*_2_)	
(iii.a) (−, −)	Same as (i)

(iii.b) (+, +)	$\left\{ {\matrix{ \displaystyle{{{a_{\bar y}^*} \over {a_{x\bar y}^*}} > {{a_{\bar yx}^*} \over {{a_x}}},} {\left( {{r_x} < 0,r_{\bar y}^* < 0} \right)} {} \cr \displaystyle{{{a_{\bar y}^*} \over {a_{x\bar y}^*}} > {{a_{\bar yx}^*} \over {{a_x}}}{\rm{\ }}} \displaystyle{{{r_{\bar y}^*} \over {{r_x}}} < {{a_{\bar yx}^*} \over {{a_x}}}} {\left( {{r_x} > 0,r_{\bar y}^* < 0} \right)} \cr \displaystyle{{{a_{\bar y}^*} \over {a_{x\bar y}^*}} > {{a_{\bar yx}^*} \over {{a_x}}}{\rm{\ }}} \displaystyle{{{r_{\bar y}^*} \over {{r_x}}} > {{a_{\bar y}^*} \over {a_{x\bar y}^*}},} {\left( {{r_x} < 0,r_{\bar y}^* > 0} \right)} \cr {} {} {} \cr } } \right.$

**Table A.4:** Parameter values used in Fig. 5.

Panel	*b*_*x*_	*by*_1_	*by*_2_	*d*_*x*_	*dy*_1_	*dy*_2_	*a*_*x*_	$\boldsymbol a_{xy_1}$	$\boldsymbol a_{xy_2}$	$\boldsymbol a_{y_1x}$	$\boldsymbol a_{y_1}$	$\boldsymbol a_{y_1y_2}$	$\boldsymbol a_{y_2x}$	$\boldsymbol a_{y_2y_1}$	$\boldsymbol a_{y_2}$	*m*
*a*	0.68	0	0.57	0.13	0.19	0.29	−0.296	−0.564	−0.805	−0.808	−0.272	−0.905	−0.406	−0.222	−0.034	3.81
*b*	0.67	0	0.65	0.4	0.17	0.37	−0.127	−0.487	−0.823	−0.933	−0.564	−0.069	0.252	−0.370	−0.09	0.95
*c*	0.55	0	0.8	0.29	0.28	0.26	−0.013	0.922	−0.956	−0.537	−0.621	−0.858	0.042	−0.448	−0.279	1.59

#### Generalizations of the role-level model

##### Two-species *n*-role model

It is straightforward to extend the two-role assumption by following the similar step presented in Section “Derivation of the role-level dynamics”. Assume that the number of roles of species *y* is *n*, and represent these roles by **y** = (*y*_1_, … , *y*_*n*_). The order of the elements of the vector corresponds to the order of occurrence of each role in the species’ life history; hence, the state of species *y* at birth is *y*_1_. All roles contributing to the growth of species *y* appear in the dynamics of *y*_1_, and the transition rate *m* connects each role. The two-species role-level dynamics become

(A.4) $$\eqalign{
  & {{dx} \over {dt}} = x\left( {{r_x} + \sum\limits_{Z \in \left\{ {x,y} \right\}} {{a_{xZ}}} Z} \right),  \cr 
  & {{d{y_1}} \over {dt}} = {y_1}\left( {{r_{{y_1}}} + \sum\limits_{Z \in \left\{ {x,y} \right\}} {{a_{{y_1}Z}}} Z} \right) + {\sum _{Y \in {\bf{y}}\backslash {y_1}}}\left( {{b_Y} + {I_Y}{a_{Yx}}x} \right){y_Y} - m{y_1},  \cr 
  & {{d{y_i}} \over {dt}} = {y_i}\left( { - {d_{{y_i}}} + {\sum _{Z \in \left\{ {x,{\bf{y}}\backslash {y_i}} \right\}}}{a_{{y_i}Z}}Z + \left( {1 - {I_{{y_i}}}} \right){a_{{y_i}x}}x} \right) + m\left( {{y_{i - 1}} - {y_i}} \right),\quad \left( {1 < i < n} \right)  \cr 
  & {{d{y_n}} \over {dt}} = {y_n}\left( { - {d_{{y_n}}} + {\sum _{Z \in \left\{ {x,{\bf{y}}\backslash {y_n}} \right\}}}{a_{{y_n}Z}}Z + \left( {1 - {I_{{y_n}}}} \right){a_{{y_n}x}}x} \right) + m{y_{n - 1}}, \cr} $$

where **y**\*y*_*i*_ = (*y*_1_, … , *y*_*i*__−__1_, *y*_*i*__+ 1_, … ,*y*_*n*_). Equation (A.4) again recovers the Lotka–Volterra equation, as in the main text:

(A.5) $$\eqalign { & {\displaystyle{{dx} \over {dt}} = x\left( {{r_x} + {a_x}x + {a_{x\bar y}}\left( t \right)\bar y} \right),} \cr & {\displaystyle{{d\bar y} \over {dt}} = \bar y\left( {{r_{\bar y}}\left( t \right) + {a_{\bar y}}\left( t \right)\bar y + {a_{\bar yx}}\left( t \right)x} \right),} \cr }$$

where ${r_{\bar y}}$, ${a_{x\bar y}}$, ${a_{\bar yx}}$, and ${a_{\bar y}}$ differ from Eq. (7) in the main text. In the *n*-role case, these parameters are described as follows:

(A.6a) $${r_{\bar y}} = \sum\limits_Y {{r_Y}} {\alpha _Y},$$

(A.6b) $${a_{x\bar y}} = \sum\limits_Y {{a_{xY}}} {\alpha _Y},$$

(A.6c) $${a_{\bar yx}} = \sum\limits_Y {{a_{Yx}}} {\alpha _Y},$$

(A.6d) $${a_{\bar y}} = \sum\limits_{Y,Y'} {\left( {{a_{YY'}} + {a_{Y'Y}}} \right)} {\alpha _Y}{\alpha _{Y'}},$$

where *α*_*Y*_ is the fraction of role *Y* $\in$ {*y*_1_, … , *y*_*n*_}, which satisfies *αy*_1_ + $\cdots$ + *αy*_*n*_ = 1. *a*_*YY*′_ is the intraspecific interaction of the heterogeneous types *Y*,*Y*′ $\in$ {*y*_1_, … , *y*_*n*_}. Hence, the population-level growth rate $r_{\bar{y}}$ and the interspecific interactions $a_{x\bar{y}}$ and $a_{\bar{y}x}$ are represented by the average value of the role-level model.

##### Two-species (*n* + *n*′)-role model

It is straightforward to integrate *n*′ roles in species *x* into the two-species *n*-role model presented above. At the population-level dynamics, the role-level dynamics again recover the Lotka–Volterra equation:

(A.7) $$\matrix{ {\displaystyle{{d\bar x} \over {dt}} = \bar x\left( {{r_{\bar x}}\left( t \right) + {a_{\bar x}}\left( t \right)\bar x + {a_{\bar x\bar y}}\left( t \right)\bar y} \right),} \cr {\displaystyle{{d\bar y} \over {dt}} = \bar y\left( {{r_{\bar y}}\left( t \right) + {a_{\bar y}}\left( t \right)\bar y + {a_{\bar y\bar x}}\left( t \right)\bar x} \right),} \cr }$$

where the total abundance of species *x* is denoted by ${\bar{x}}$ = *x*_1_ + $\cdots$ + *x*_*n*′_ and the growth rate $r_{\bar{x}}(t)$ and intraspecific competition $a_{\bar{x}}(t)$ are time-dependent. These coefficients are analogous to Eq. (A.6). The main difference is in $a_{x\bar{y}}$ and $a_{y\bar{x}}$ as a result of the existence of *n*′ roles in species *x*. These terms are described by

(A.8a) $${r_{\bar x}} = \sum\limits_X {{r_X}} {\alpha _X},$$

(A.8b) $${r_{\bar y}} = \sum\limits_Y {{r_Y}} {\alpha _Y},$$

(A.8c) $${a_{\bar x\bar y}} = \sum\limits_{X,Y} {{a_{XY}}} {\alpha _X}{\alpha _Y},$$

(A.8d) $${a_{\bar y\bar x}} = \sum\limits_{X,Y} {{a_{YX}}} {\alpha _X}{\alpha _Y},$$

(A.8e) $${a_{\bar x}} = \sum\limits_{X,X'} {\left( {{a_{XX'}} + {a_{X'X}}} \right)} {\alpha _X}{\alpha _{X'}},$$

(A.8f) $${a_{\bar y}} = \sum\limits_{Y,Y'} {\left( {{a_{YY'}} + {a_{Y'Y}}} \right)} {\alpha _Y}{\alpha _{Y'}},$$

where the notation associated with *X* is analogous to that of *Y*. For example, *α*_*X*_ is the fraction of role *X* $\in$ {*x*_1_, … , *x*_*n*′_}, which satisfies *αx*_1_ + $\cdots$ + *αx*_*n*′_ = 1.

##### N-species n-role model

The generalization to an *N-*species *n-*role model is also straightforward, where *n* = (*n*_1_, … , *n*_*N*_) represents the set of all species roles. Let *x*_*i*_ be species *i*, and consider the population-level dynamics given by the *N-*species Lotka–Volterra equation

(A.9) $$\matrix{ {\displaystyle{{d{{\bar x}_i}} \over {dt}} = {{\bar x}_i}\left( {{r_{{{\bar x}_i}}}\left( t \right) + {a_{{{\bar x}_i}}}\left( t \right){{\bar x}_i} + \sum\limits_{{x_j} \ne {x_i}} {{a_{{{\bar x}_i}{{\bar x}_j}}}} \left( t \right){{\bar x}_j}} \right),} \cr {} & {} \cr }$$

where

(A.10a) $${r_{{{\bar x}_i}}} = \sum\limits_{{X_i}} {{r_{{X_i}}}} {\alpha _{{X_i}}},$$

(A.10b) $${a_{{{\bar x}_i}{{\bar x}_j}}} = {\sum _{{X_i},X\backslash {X_i}}}{a_{{X_i}X}}{\alpha _{{X_i}}}{\alpha _X},$$

(A.10c) $${a_{{{\bar x}_i}}} = \sum\limits_{{X_i},{{X'}_i}} {\left( {{a_{{X_i}{{X'}_i}}} + {a_{{{X'}_i}{X_i}}}} \right)} {\alpha _{{X_i}}}{\alpha _{{{X'}_i}}},$$

in which ${X_i} \in \left\{ {{x_{i1}}, \cdots ,{x_{i{n_1}}}} \right\}$, $X \in \left\{ {{x_{11}}, \cdots ,{x_{1{n_1}}}, \cdots {x_{N1}}, \cdots ,{x_{N{n_N}}}} \right\}$, and

$X\backslash {X_i} \in \left\{ {{x_{11}}, \cdots ,{x_{1{n_1}}}, \cdots {x_{i - 1,1}}, \cdots ,{x_{i - 1,{n_{i - 1}}}},{x_{i + 1,1}}, \cdots ,{x_{i + 1,{n_{i + 1}}}}, \cdots {x_{N1}}, \cdots ,{x_{N{n_N}}}} \right\}$.

#### Specific form of the role-level model

##### From inferior competitor to superior competitor

From Eq. (5), the models under different role-shift scenarios can be derived. When the role-shift is from inferior competitor to superior competitor,

(A.11) $$\eqalign{
  & {{dx} \over {dt}} = x\left( {{r_x} + {a_x}x + {a_{x{y_1}}}{y_1} + {a_{x{y_2}}}{y_2}} \right),  \cr 
  & {{d{y_1}} \over {dt}} = {y_1}\left( {{r_{{y_1}}} + {a_{{y_1}}}{y_1} + {a_{{y_1}{y_2}}}{y_2} + {a_{{y_1}x}}x} \right) + {b_{{y_2}}}{y_2} - m{y_1},  \cr 
  & {{d{y_2}} \over {dt}} = {y_2}\left( { - {d_{{y_2}}} + {a_{{y_2}x}}x + {a_{{y_2}{y_1}}}{y_1} + {a_{{y_2}}}{y_2}} \right) + m{y_1}, \cr} $$

where, by definition, $|{a_{x{y_1}}}| < |{a_{{y_1}x}}|$and $|{a_{x{y_2}}}| > |{a_{{y_2}x}}|$ are satisfied.

From competitor to predator

(A.12) $$\eqalign{
  & {{dx} \over {dt}} = x\left( {{r_x} + {a_x}x + {a_{x{y_1}}}{y_1} + {a_{x{y_2}}}{y_2}} \right),  \cr 
  & {{d{y_1}} \over {dt}} = {y_1}\left( { - {d_{{y_1}}} + {a_{{y_1}}}{y_1} + {a_{{y_1}{y_2}}}{y_2} + {a_{{y_1}x}}x} \right) + {a_{{y_2}x}}x{y_2} - m{y_1},  \cr 
  & {{d{y_2}} \over {dt}} = {y_2}\left( { - {d_{{y_2}}} + {a_{{y_2}{y_1}}}{y_1} + {a_{{y_2}}}{y_2}} \right) + m{y_1}. \cr} $$

I assume that species *y* is a specialist predator, and the population growth comes from the predation of species *x*.

From prey to predator

(A.13) $$\eqalign{

   & \displaystyle{}{{dx} \over {dt}} = x\left( { - {d_x} + {a_x}x + {a_{x{y_1}}}{y_1} + {a_{x{y_2}}}{y_2}} \right), \cr
   & \displaystyle{{d{y_1}} \over {dt}} = {y_1}\left( { - {d_{{y_1}}} + {a_{{y_1}}}{y_1} + {a_{{y_1}{y_2}}}{y_2} + {a_{{y_1}x}}x} \right) + {a_{{y_2}x}}x{y_2} - m{y_1}, \cr
   & \displaystyle{}{{d{y_2}} \over {dt}} = {y_2}\left( { - {d_{{y_2}}} + {a_{{y_2}{y_1}}}{y_1} + {a_{{y_2}}}{y_2}} \right) + m{y_1},}$$

where species *x* is simultaneously the predator of *y*_1_ and the prey of *y*_2_. Additionally, species *x* is a specialist predator, and so population growth occurs by consuming *y*_1_. The growth rate of species *X* is denoted by *r*_*X*_ = − *d*_*X*_.

### Numerical error search

In the population-level dynamics (Eq. (6)) in the main text, the isolines at equilibrium are not unique for cases of bistability. This is the source of the mismatch in equilibrium values between the role-level and population-level dynamics. To check the match between the two models, 10^5^ parameter sets were randomly generated and the equilibrium values of the role-level and population-level dynamics were checked for each of the parameters. I counted a parameter set as mismatch if the equilibrium values satisfied with $|{x^*} - x_{pop}^*| + |y_1^* + y_2^* - {\bar y^*}| > {10^{ - 3}}$ where $x_{pop}^*$ is the equilibrium of species *x* in the population-level model. Each parameter sets were examined with two initial conditions to check bistability. The parameter sets were generated in the following manner:

(B.1) $$\matrix{ {{b_Z}\sim U\left[ {0,1} \right],{d_Z}\sim U\left[ {0,0.5} \right],{a_Z}\sim U\left[ { - 1,0} \right],|{a_{ZZ}}|\sim U\left[ {0,1} \right],m\sim U\left[ {0,20} \right],} \cr }$$

where *Z* = {*x,y*_1_*,y*_2_}, *a*_*ZZ*_ represents all possible heterogeneous pairs of {*x,y*_1_*,y*_2_}, and *U*[*a,b*] is a uniform distribution over the range [*a,b*]. The role-shift scenario was chosen at random, and the signs of *a*_*ZZ*_ were chosen accordingly. I found 238 parameter sets (ca. 0.24%) that caused inconsistent equilibrium values. All these cases led to a bistability condition in the population-level model, but the role-level dynamics instead converged to a stable equilibrium for cases (i), (ii), and (iii.a), or to one of the equilibria regardless of stability for case (iii.b) in Table A.4.

## Supplemental Information

10.7717/peerj.13315/supp-1 Supplemental Information 1 Numerical calculations of role-level and population dynamics are implemented by the code.

10.7717/peerj.13315/supp-2 Supplemental Information 2 A numerical error search was conducted by the code.

10.7717/peerj.13315/supp-3 Supplemental Information 3 Figure 2 was generated using the code.

## Additional Information and Declarations

### Data Availability

The following information was supplied regarding data availability: The code are available in the Supplemental Files.
